# Dynamics of Gastro-Intestinal Strongyle Parasites in a Group of Translocated, Wild-Captured Asiatic Wild Asses in Kazakhstan

**DOI:** 10.3389/fvets.2020.598371

**Published:** 2020-12-11

**Authors:** Diana S. Gliga, Natalia Petrova, John D. C. Linnell, Albert R. Salemgareyev, Steffen Zuther, Chris Walzer, Petra Kaczensky

**Affiliations:** ^1^Department of Interdisciplinary Life Sciences, University of Veterinary Medicine Vienna, Vienna, Austria; ^2^Moscow State Academy of Veterinary Medicine and Biotechnology, Moscow, Russia; ^3^Norwegian Institute for Nature Research, Trondheim, Norway; ^4^Association for the Conservation of Biodiversity of Kazakhstan, Nur-Sultan, Kazakhstan; ^5^Frankfurt Zoological Society, Frankfurt, Germany; ^6^Wildlife Conservation Society, New York, NY, United States

**Keywords:** Asiatic wild ass, *Equus hemionus kulan*, gastro-intestinal parasites, reintroduction, fecal egg count, strongyles

## Abstract

Asiatic wild ass (Kulan, *Equus hemionus*) population range and numbers became severely reduced and a reintroduction project is currently aiming to re-establish a population in the Central Steppe of Kazakhstan. Pre-emptive deworming is often recommended for equid translocations but eliminating parasites prior to translocation could cause disruptions in a balanced host-parasite relationship, adding an additional stressor to an already stressful intervention involving capture, transport, and adaptation to a new environment. Following a disease risk assessment, we decided against pre-emptive deworming and focused on monitoring the first group of nine translocated kulan in a large acclimatization enclosure prior to release. Over the 5-month acclimatization period, we regularly collected fecal samples and analyzed the shedding intensity of gastro-intestinal parasite eggs, obtained time budgets through behavioral observations, and visually assessed body condition. We identified strongyles (*Strongylinae* and *Cyathostominae*) and pinworms (*Oxyuris equi*) in fecal samples. All individuals shed strongyle eggs and two of the nine individuals had higher shedding intensities, but rarely reached levels for which deworming is recommended. All kulan appeared healthy throughout the observation period, aggressive interactions were very rare, and time budgets were very similar and dominated by feeding. Our results suggest that in translocation projects where the risk of introducing new parasites is minimal, pre-emptive treatment in wild equids can be replaced with non-invasive monitoring during the acclimatization period. We acknowledge that the small number of kulan, the large size of the enclosure, and the low temperatures during the animals stay in the acclimatization enclosure may all have reduced infestation pressure.

## Introduction

Conservation translocations aim at restoring species and ecosystem functioning, but require a careful risk assessment. One of the risks is that the new arrivals can transfer infectious diseases and parasites, which may have potentially detrimental effects for wildlife, livestock or humans in the release area (1, 2). However, autochthonous parasites must be viewed as both a natural component of biodiversity worthy of conservation and a key ecological component of a normal ecosystem. Furthermore, health is not a binary state, and wild animals live in a dynamic state with a large number of commensals, symbionts, and parasites which support digestion while shaping and challenging the immune system (3–5).

Gastro-intestinal parasites, particularly strongylids, are common in domestic and wild equids (6, 7). Strongylids can cause serious disease in domestic equids if not controlled with antihelmintics (8). Symptoms of strongylid-induced colics in equids can range from behavioral changes such as head turning to flank, lying down, and rolling, to diarrhea (9). Infestations with other gastro-intestinal parasites such as *Oxyuris equi* are known to cause excessive tail rubbing, those with *Gasterophilus spp*. to cause rectal prolapse, and those with *Parascaris equorum* to cause respiratory distress (9, 10). Free-living wild equids are likely adapted to their parasites and rarely show clinical signs (7, 11). However, high stress management interventions such as capture and transport could weaken the immune system (12) and confinement to an adaptation enclosure over an extended time with conspecifics could result in the exposure to infectious parasite due to the accumulation of feces (13), both of which could result in unusually high parasite burdens. Fecal examination of endoparasites and treatment are generally recommended for equid reintroductions (14), but eliminating parasites prior to translocation could cause disruptions in a balanced host-parasite relationship, adding an additional stressor to an already stressful intervention involving capture, handling, transport, and adaptation to a new environment (12, 15, 16).

Behavioral observations and time budgets are commonly used to assess animal welfare and wild equids can be expected to spend the majority of their time grazing and show high group synchronization (17, 18). Reduced feeding time, strong deviation from group behavior, high levels of aggressive interactions, or signs of disease are indicative of welfare problems which may also be associated with high parasite burdens (19). High parasite burden can also negatively affect body condition (20, 21), suggesting that behavioral observations should be combined with body condition scoring (22).

In Kazakhstan, Asiatic wild asses (kulan, *Equus hemionus kulan*) were eradicated by the 1930s (23, 24). Reintroductions, starting in the 1950s re-established the species in two protected areas, Barsa-Kelmes State Nature Reserve (SNR) in south-western, and Altyn Emel National Park (NP) in south-eastern Kazakhstan (25). However, kulan have not yet reclaimed more than 1% of their former range in Kazakhstan and have remained absent from the Central Steppes. To increase kulan population size and range, and restore the original herbivore assemblage of the Central Steppes, a kulan reintroduction project was initiated in 2016 (26).

Domestic equids (horses and donkeys) are distributed throughout Kazakhstan and are widely traded and transported across administrational borders. Domestic equids occur at both sources for kulan translocations, Altyn Emel NP and Barsa-Kelmes SNR, and at the receiving end on the Central Steppes. Consequently, the risk of introduction of new infectious agents to the release area or exposure of translocated kulan to new infectious agents was rated as very low (26). Furthermore, previous field studies have shown that gastro-intestinal parasites are a normal component of free-ranging Asiatic wild asses in Mongolia, where they share the range with reintroduced Przewalski's horses (*Equus ferus przewalskii*) and domestic horses (7).

The aim of this study was to determine if pre-emptive de-worming in translocated wild kulan in a large acclimatization enclosure could be replaced with non-invasive monitoring methods. Our data provide the first time series on gastro-intestinal parasites, behavior, and body condition for a small group of wild kulan under semi-natural conditions following a highly invasive management intervention.

## Methods

### Study Animals

Kulan were captured in Altyn Emel NP using a large capture corral (27). To transfer kulan into transport boxes, selected animals were anesthetized following standard procedures (28, 29). All individuals were vaccinated against rabies and anthrax during handling, but did not receive antiparasitic treatment. Kulan were marked with numbered plastic ear tags for individual identification and adult mares were additionally equipped with GPS-satellite collars (VERTEX Lite Iridium, Vectronic Aerospace, Berlin, Germany) to facilitate post-release monitoring.

On 24 October 2017, nine kulan, four adult mares, four foals and one subadult stallion, were successfully transported via helicopter over 1,300 km from Altyn Emel NP to the Central Steppes (30). The animals were released into a 55 ha adaptation enclosure at Alibi field station (N 49.3067/E 64.5164) located on the edge of Altyn Dala Nature Reserve (NR).

The nine kulan were monitored from arrival on 24 October 2017 until 26 March 2018 (Table 1). They were supplied with water in troughs until end of November 2017 (after which snow covered the ground) and *ad-libitum* hay in a shelter where kulans preferred to stay over-night. Dung was removed from the shelter almost daily to reduce the chance of contamination. The group was released into the wild with the beginning of spring on 4 April 2018 (31).

**Table 1:** Kulan monitored at the Alibi research station from arrival on 24 October 2017 until 26 March 2018.

**Kulan ID^1^**	**Sex**	**Age group**	**Age**	**Mare – foal associations**	**Eartag**	**Collar #^2^**	**Collar ID^3^**
AF5	f	adult	7	FM8	3	5	26860
AF17	f	adult	7	FF7	4	17	26176
AF4	f	adult	6	FM11	5	4	26855
AF9	f	adult	5	no foal^4^	6	9	26859
FF7	f	foal	0.5		7		
FM8	m	foal	0.5		8		
FF0	f	foal	0.5	no mare^5^	none		
FM11	m	foal	0.5		11		
SM12	m	subadult	3		12		

1 *ID bases on sex and collar/eartag ID*.

2 *Number written on collar*.

3 *Iridium number*.

4 *No milk in the udder and the absence of a foal suggests that mare was without a foal at the time of capture*.

5 *This foal got separated from its mother during capture. AF, Adult female; SM, Subadult male; FF, Foal female; FM, Foal male*.

### Collection and Processing of Individual Fecal Samples

To collect fecal samples, we observed kulan with binoculars and/or a spotting scope until defecation of the target kulan. To ascertain that samples could be assigned to a specific individual, only pellets dropped in a heap by a standing targeted kulan were sampled. Sampling of one individual kulan normally took between 1 and 2 h but became easier once kulan tolerated observers at shorter distances. From the fresh dung pile, we took random samples from the inside of several boli (to avoid contamination with soil) amounting to ca. 80 g. We placed samples into washed and disinfected jars for further processing in the field laboratory later the same day. There, each fecal sample was thoroughly mixed and five subsamples were taken for analysis.

To determine presence, quantity, and identity of gastro-intestinal parasites we used: (1) the McMaster egg counting method [4 g fecal sample, detection threshold 50 eggs per gram (EPG)] to measure the amount of parasite eggs passed in feces [fecal egg count (FEC)], (2) a combined sedimentation-flotation method (CSFM, 5 g fecal sample) and Benedek's sedimentation method (20 g fecal sample) to qualitatively detect parasites at a higher sensitivity, (3) the Baerman method (20 g fecal sample) to isolate lung worm larvae, and (4) visual inspection under a microscope (20 g fecal sample) for third stage strongyle larva differentiation. These methods were performed as described by Schnieder (32), with one exception, adouble layered gauze was used instead of sieves. The sugar solution for the McMaster and flotation methods was prepared at a 1.27 specific gravity.

Between November 2017 and March 2018, we sampled each individual kulan at least once during seven 10-day sampling blocks, providing 63 samples suitable for analysis (Supplementary Table 1). Ambient temperature during field work averaged –10°C and ranged from +18.2°C to −32.5°C (Davis Vantage Pro 2 Basic; Supplementary Figure 1). Differences between individual egg shedding over time were analyzed with a Kruskal-Wallis Test.

### Animal Observations

We checked kulan daily, visually assessing body condition on a scale from 0 (very thin, ribs and point of pelvis very visible, rump concave, skin below backbone sunken) to 5 (very fat, ribs and point of pelvis not seen, rump very convex) (22), and checking for signs of injuries or disease.

We recorded individual time budgets, using instantaneous scan sampling during one to four 1-h long observation periods each day (33). Behavioral scans for main behavioral categories were initially performed at 15 min intervals (from 30th Oct 2017 to 13th Feb 2018) and later at 5 min intervals (from 14th Feb to 26th March 2018).

We focused on four main behaviors to obtain individual time budgets:

**Feeding:** kulan stand or walk slowly with their heads below shoulder level, and biting, chewing or swallowing food during walking with their muzzles close to the ground.

**Walking**: kulan walk and **running**: kulan run (or trot) with the head at or above the shoulder level.

**Standing:** kulan standing motionless for a longer time (over 1 min) with the head at or above shoulder level.

**Alert:** kulan standing, but exhibiting a rigid body posture with head upright, ears pointed forward, and eyes open and focused.

**Lying down:** kulan lying on the ground.

**Other:** all other behavior.

We additionally recorded all events of mutualistic (play, grooming, suckling) and antagonist (chasing away, biting, kicking, fighting) interactions during the entire observation hour.

In total, we conducted 271 sessions of behavioral observations of 1 hour each. After removal of incomplete observations (*n* = 10) due to field constraints and observations where individual identification was not possible (*n* = 17), 244 observation sessions remained for further analyses.

## Results

### Parasitology

**Figure 1:**
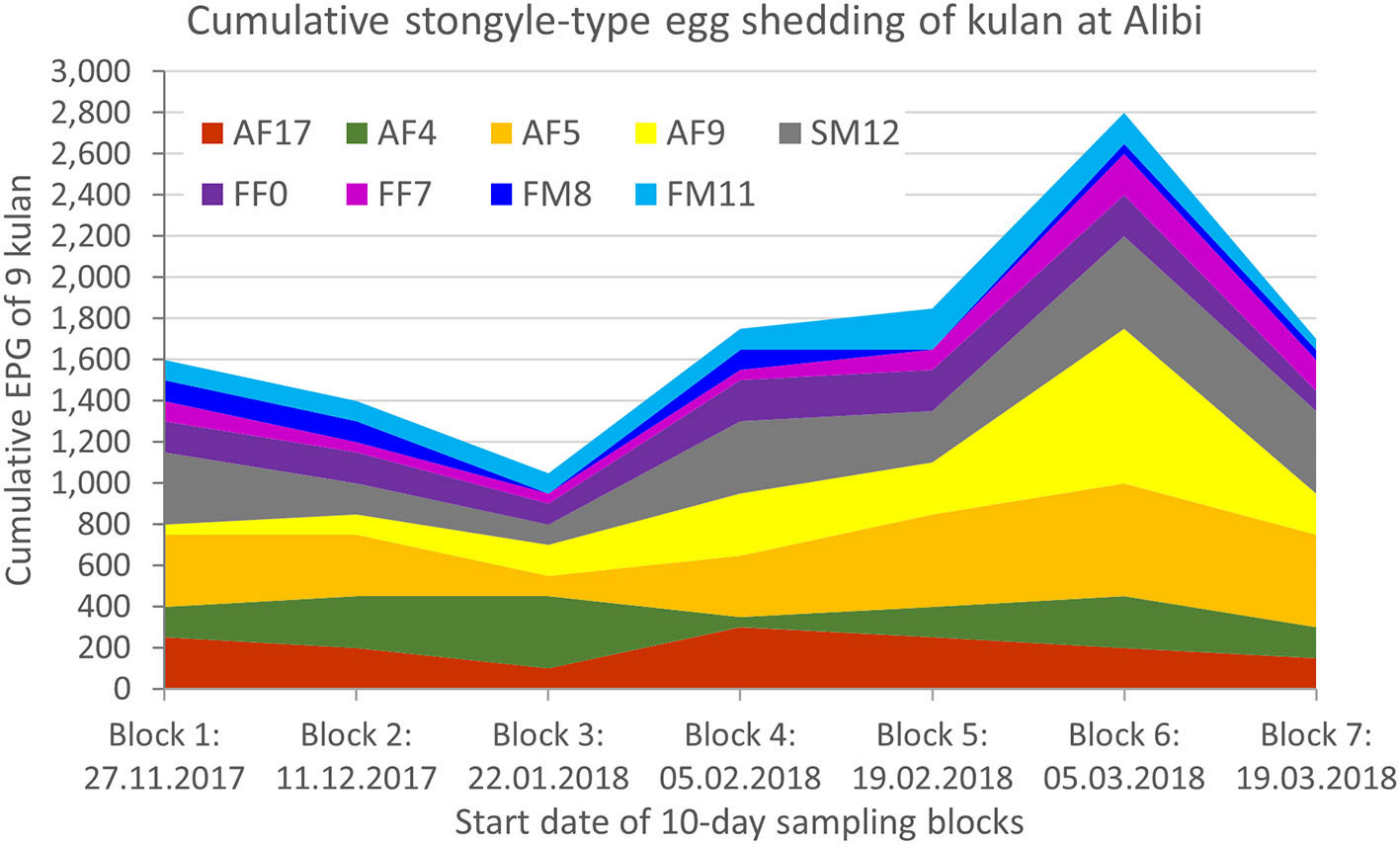
Individual and cumulative strongyle-type egg shedding over time. EPG, eggs per gram.

All individuals shed strongyle-type eggs (*Strongylinae* and *Cyathostominae*). In two samples FEC was null, but strongyle eggs were detected qualitatively with the CSFM. The mean strongyle-egg output was 192.9 ± 140.8 EPG over the whole sampling period. We recorded the highest shedding intensity (311.1 ± 224.7 EPG) in sampling block 6 (5-14.03.2018) and the lowest (116.7 ± 96.8 EPG) in sampling block 3 (22-31.01.2018) (Table 2, Figure 1).

**Table 2:** Strongyle-type egg shedding by sampling block and individual.

**Sampling unit**	**Time period**	**Mean EPG**	**SD**	**Median**
**Group values over time**				
Block 1	27.11.17–06.12.17	177.8	112.1	150
Block 2	11.12.17–20.12.17	155.6	80.8	150
Block 3	22.01.18–31.01.18	116.7	96.8	100
Block 4	05.02.16–14.02.18	194.4	121.0	200
Block 5	19.02.18–28.02.18	205.6	123.6	200
Block 6	05.03.18–14.03.18	311.1	224.7	200
Block 7	19.03.18–25.03.18	188.9	143.1	150
All	27.11.17–25.03.18	192.9	140.8	150
**Individual values**				
AF17	27.11.17–25.03.18	207.1	67.3	200
AF4		192.9	97.6	150
AF5		357.1	145.6	350
AF9		257.1	233.5	200
SM12		292.9	130.5	350
FF0		157.1	45.0	150
FF7		100.0	57.7	100
FM8		57.1	45.0	50
FM11		114.3	47.6	100

*AF, Adult female; SM, Subadult male; FF, Foal female; FM, Foal male; SD, Standard deviation; Raw data of fecal egg counts is available in Supplementary Table 1*.

Egg shedding over the entire sampling period differed significantly among individuals (Independent-Samples Kruskal-Wallis Test, *p* < 0.001; Table 2, Figure 1). Two potential high shedders, which usually had higher values than the other individuals, were identified in the kulan group. These were the subadult male SM12 (292.9 ± 130.5) and the adult female AF5 (357.1 ± 145.6).

We detected *Oxyuris equi* eggs in the feces of seven of the nine kulan (78%) using the McMaster counting method or the CSFM. On two occasions, adult worms were observed on the surface of feces dropped by foals. The identity of these worms was later confirmed as *O. equi* at the Institute of Parasitology at the University of Veterinary Medicine, Vienna. Kulan did not show signs of perineal pruritus associated with clinical pinworm infection.

Due to high variations in indoor temperatures in the basic field laboratory, larval culture yield was inconsistent, and the output was only analyzed qualitatively. Some L3 larvae of small and large strongyles were identified in the harvest of the larval culture, but most larvae were either underdeveloped or showed signs of autolysis, which made the morphological identification impossible.

No lung worm larvae were detected with the Baerman method.

### Behavioral Observation and Body Scoring

**Figure 2:**
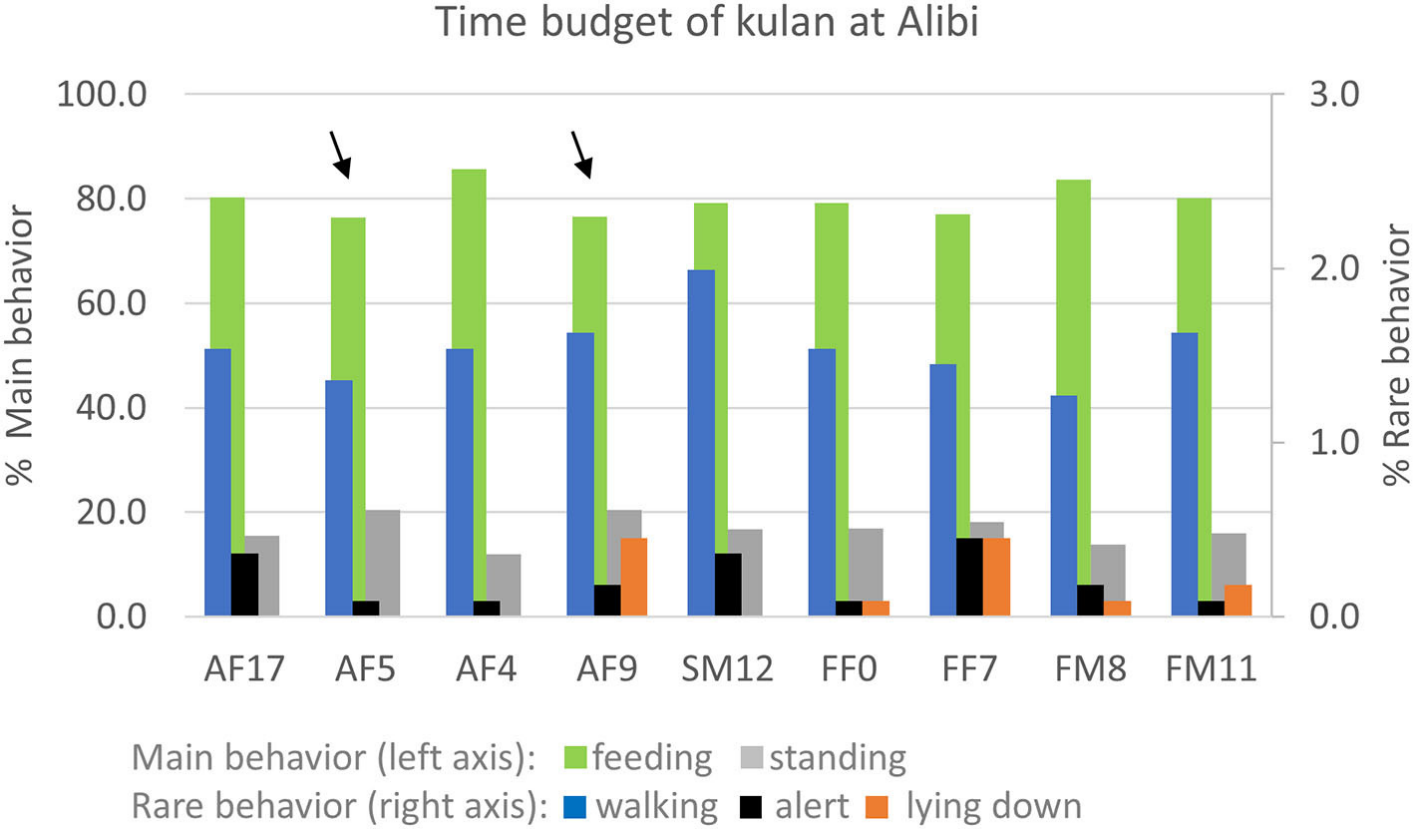
Time budget of nine kulan in the 55 ha acclimatization enclosure at Alibi from 6th Nov 2017 to 26th Mar 2018. Arrows mark the two individuals that had consistently higher egg shedding values.

Behavioral observations did not reveal any major differences in the overall time budget of the nine kulan. All kulan were primarily feeding (72–80%) and standing (17–25%), whereas behaviors such as walking (2–2.4%), alert (0.1–0.8%), and lying down (0.1–3.4%) were rare or very rare (Figure 2). The two kulan, SM12 and AF5, that had higher FEC, did not differ in a consistent way in their time budget from the other animals.

The nine kulan formed a rather cohesive group and all individuals moved together most of the time. Even the motherless foal FF0 was fully integrated into mutualistic behavior with other foals and adults; the only difference was that motherless foal FF0 was not allowed to suckle. Two foals (FF7, FM11) were nursed over the entire monitoring period, while one foal (FM8) was last seen suckling on 11th Dec 2017.

Aggressive interactions were extremely rare and consisted of brief momentary reactions like biting, kicking or chasing away. Of 19 aggressive instances recorded, 5 were initiated by AF5 and 6 by SM12, with the remaining 7 kulan initiating 0–3 aggressive interactions each.

**Figure 3:**
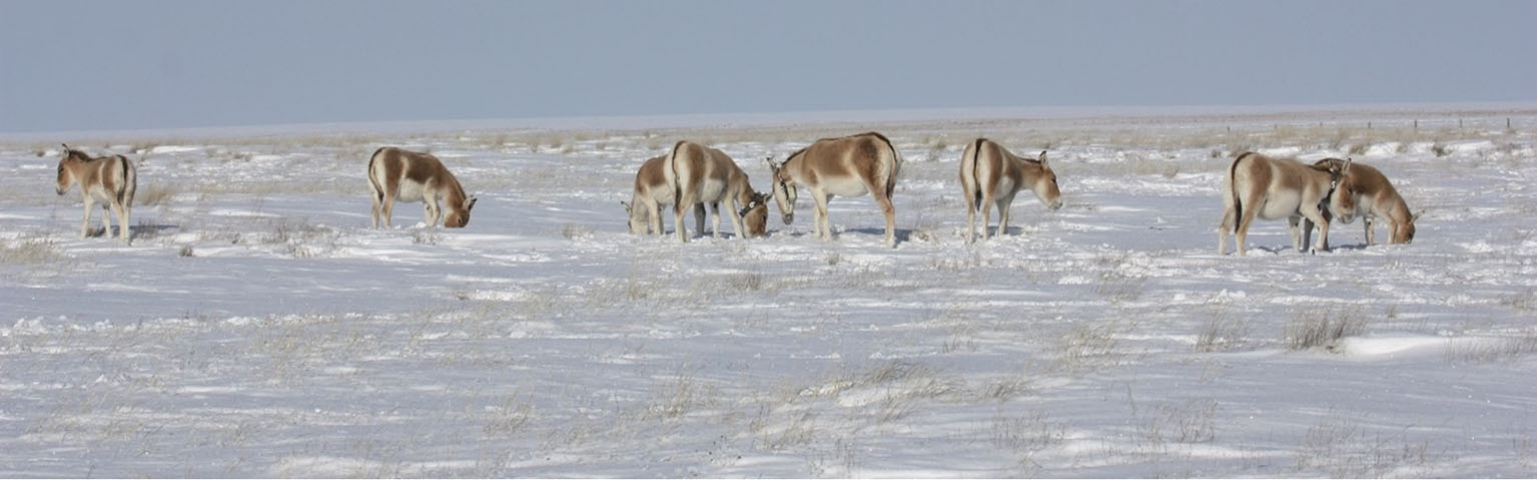
Translocated kulan digging snow to feed on the natural pasture in the acclimatization enclosure in Alibi in January 2018. Photo: D. Gliga and N. Petrova.

No kulan showed signs of poor body condition, disease, or injuries apart from occasional minor cuts and bruises (Figure 3). We estimated a body condition score of 2 (fair) to 3 (good) in all kulan over the entire monitoring period.

## Discussion

### Parasites

The observed 100% prevalence of strongyles was expected as they are the most common endoparasites in wild herbivores. Strongyles have been previously documented in wild kulan in Mongolia, and in domestic horses in Kazakhstan (7, 34). Strongyles have been found in coprolites of Kazakh domestic horses dating back as early as the 3rd century BC (35), which suggests that wild and domestic equids have shared and co-evolved with some of these parasites throughout history. Species identification was hampered by the lack of an incubator as leaving larval cultures for 14 days at unstable room temperature was not enough for the development of L3 larvae, while extending this time increased the risk of mold contamination.

Two individuals (subadult stallion SM12 and adult female AF5) had higher FECs, but largely stayed below the 500 FEC threshold for high egg shedders used for domestic horses (36). The values were also lower than those observed in wild kulan in Mongolia [mean: 815, (7)] and in the lower range of values found in other free-ranging wild and feral equids (where values are often >800 FECs; Supplementary Table 2). Threshold values of FEC for selective antiparasitic treatment remain under debate, but tend to be somewhere around 200–500 EPG for domestic horses and wild equids in a zoo environment, respectively (36–38). Although individual values of kulan did occasionally exceed 400 EPG, none of the kulan showed any signs of gastro-intestinal discomfort (8) during the monitoring period.

The overall decrease in fecal egg count in mid-winter and the subsequent increase at the beginning of March is in line with a spring rise described in other wildlife such as red deer (*Cervus elaphus*), other wild equids such as Przewalski's horses (*Equus ferus przewalskii*), and in grazing ponies (39–41). The small number of kulan and the limited monitoring period does not allow us to draw any robust conclusions about seasonality.

FECs should be interpreted carefully because egg shedding is not the best estimate for parasite burden and there is often no correlation between egg counts and adult worm counts (42). However, in a study in domestic horses, FECs under 500 EPG did correspond to significantly lower adult worm loads (43). Moreover, adult worms are for the most part not responsible for disease, in contrast to the larval stages (e.g., larval cyathostominosis caused by excystment of L4 small strongyle larvae, and verminous arteritis and tissue damage caused by migrating large strongyle larvae) (42). We therefore believe that monitoring FEC is a valid measure to assess parasite load relevant for equid health but acknowledge that the relationship between parasite load and egg shedding pattern should be investigated further.

The detected pinworm prevalence was 78% (7/9), but most likely all animals were infected given that they lived in a small group in close contact. Parasite egg shedding is reduced in cold winters and hot summers when conditions outside the host are not favorable for larval development and transmission of the infectious stage, so many parasites undergo hypobiosis, a form of arrested development in the host (8, 44). This could explain the small diversity and reduced egg output of gastro-intestinal parasites detected in our kulan during winter.

### Behavior and Body Condition

Behavioral observations did not detect clinical signs of strongylid-induced colics, which is in line with FEC values mostly being below the threshold for clinical signs in domestic horses and the fact that wild equids and kulan in particular seem to be adapted to relative high parasite burdens [(7), Supplementary Table 2]. We are confident that our behavioral observations would have picked up on animal welfare issues, including signs of acute strongylosis, because the animals got used to the observers quickly and tolerated their presence at around 100 m without being disturbed.

We suspect that AF5, which had higher FECs, potentially experienced more stress because she weaned her foal earlier in winter, when compared to the two other mares that nursed their foals over the entire monitoring period. However, given the lack of causal proof and the anecdotal nature of our observation, we cannot conclude that the early weaning was a consequence of higher parasite burden, nor that stress caused this higher parasite burden (19). The two independent foals (FF0, FM8) did not appear negatively affected by the absence of nursing and maternal care. Although it is preferable to translocate mares with foals, this case shows that motherless foals from 5 to 6 months of age can survive the first winter feeding on their own when integrated in a group.

Determining body condition in kulan was difficult because the classification schemes developed for domestic ponies (22) did not align well with kulan due to the different body shape and the very thick winter coat. Body condition is reported to be negatively correlated with parasite burden in feral horses, when parasite burden is very high (21). Our general impression was that kulan body score did not change significantly over the observation period, which is consistent with mostly low FEC and the absence of a consistent trend in FEC for any of the kulan. However, more research is needed to identify a potential direct effect of FEC on body condition under natural settings.

## Conclusion

Translocations should balance the preservation of the entire host-parasite complex against the risk of accidentally introducing novel parasites which could prove harmful to biodiversity or local economy of the release site. Disease risk assessment suggested a very low chance of introducing new gastro-intestinal parasites when translocating wild kulan within Kazakhstan. The recorded low FEC values, lack of clinical signs of strongylosis, and lack of behaviors suggesting discomfort, stress, or a strong deviation from group behavior in the first group of translocated wild kulans supported our decision to not pre-emptively deworm wild kulan prior to transport. Our method is non-invasive, relatively simple, and inexpensive in respect to equipment, but is time consuming and can be challenging under adverse weather conditions. We acknowledge that the small number of kulan, the large size of the enclosure, and the low temperatures during the animals stay in the acclimatization enclosure may all have reduced infestation pressure. Although our results are quite promising they should be interpreted with caution and need further proof of concept. For the kulan reintroduction project we suggest to use the same approach in future transports and restrict anti-parasite treatment to animals which have unusually high FEC, continuously increasing FEC, or show clinical signs of gastro-intestinal parasites.

## Data Availability Statement

The original contributions presented in the study are included in the article/Supplementary Materials, further inquiries can be directed to the corresponding author/s.

## Ethics Statement

The animal study was reviewed and approved by the Committee of Forestry and Wildlife of the Ministry of Ecology, Geology, and Natural Resources of Kazakhstan (permit 17-2-18/1613 dated 17.10.2017), the Kostanay oblast (permit 17-2-18/1613 dated from 17.10.2017) and Altyn Emel National Park and Altyn Dala Nature Reserve (agreement between the protected areas). Captures, animal handling, and transport were performed in accordance with relevant guidelines and regulations. The ethic commission at the University of Veterinary Medicine Vienna was informed and provided general consent. The IUCN Equid and Reintroduction Specialist reviewed the feasibility study and provided letters of support for the reintroduction project.

## Author Contributions

PK designed and supervised the work. DG, AS, and SZ planned and organized field work. DG and NP performed all field work and organized and analyzed the data. DG wrote the first draft of the manuscript. All authors commented on the draft and approved the final submission of the manuscript.

## Conflict of Interest

The authors declare that the research was conducted in the absence of any commercial or financial relationships that could be construed as a potential conflict of interest.
