# Supplementary material for: Dynamics of Gastro-Intestinal Strongyle Parasites in a Group of Translocated, Wild-Captured Asiatic Wild Asses in Kazakhstan
Source: Front Vet Sci. 2020 Dec 11;7:598371. doi: 10.3389/fvets.2020.598371 (PMC7759666; doi:10.3389/fvets.2020.598371)
Supplement: Supplementary file 1 [file Data_Sheet_1.pdf]

Supplementary Material

1 Supplementary Tables

Supplementary Table S1. Strongyle-type egg shedding over time and by individual.

**Kulan ID**[1]	**Block 1: 27.11.2017 – 06.12.2017**	**Block 2: 11.12.2017 – 20.12.2017**	**Block 3: 22.01.2018 – 31.01.2018**	**Block 4: 05.02-2018 – 14.02.2018**	**Block 5: 19.02.2018 – 28.02.2018**	**Block 6: 05.03.2018 – 14.03.2018**	**Block 7: 19.03.2018 – 25.03.2018**	**Sum**
AF17	250	200	100	300	250	200	150	1450
AF4	150	250	350	50	150	250	150	1350
AF5	350	300	100	300	450	550	450	2500
AF9	50	100	150	300	250	750	200	1800
SM12	350	150	100	350	250	450	400	2050
FF0	150	150	100	200	200	200	100	1100
FF7	100	50	50	50	100	200	150	700
FM11	100	100	100	100	200	150	50	800
FM8	100	100	0	100	0	50	50	400
Sum	1600	1400	1050	1750	1850	2800	1700	

[1]AF=Adult female, SM=Subadult male, FF= Foal female, FM= Foal male.

Supplementary Table S2. Strongyle fecal egg count values in free-ranging wild and feral equids.

Species	Country	Facility	Strongylid FEC	Source
Khulan	Mongolia - Gobi	Free-ranging	mean: 815	Painer et al. 2011
Przewalski's horse	Mongolia - Gobi	Free-ranging	mean: 199	Painer et al. 2011
Przewalski's horse	Ukraine	Fences semi-reserve	mean: 1016.7<br>range: 375-2075	Kuzmina 2009
Przewalski's horse	Russia - Orenburg	Fences semi-reserve	range: 550 - 2600	Kuzmina 2017
Plains zebra	Tanzania - Serengeti	Free-ranging	more than 1100 (adults)	Seeber et al. 2020
Plains zebra	Kenya - Laikipia & Samburu	Free-ranging	mean: ca. 2300	Rubenstein 2010
Grevy's zebra	Kenya - Laikipia & Samburu	Free-ranging	mean: ca. 1200	Rubenstein 2010
Domestic hores	Mongolia - Gobi	Free-ranging	mean: 674	Painer et al. 2011
Feral horses	USA - Sable Island	Free-ranging	mean: 1543	Debeffe et al. 2016
Feral horses	USA - Sable Island	Free-ranging	mean: 689 (spring) mean: 1105 (summer)	Jenkins et al. 2020
Feral horses	USA - Shackelford Banks	Free-ranging	range: 50-76,875	Rubenstein and Hohmann 1989
Feral horses	New Zealand - Kaimanawa	Free-ranging	more than 1700	Pomroy et al. 1995

References

Debeffe, L., McLoughlin, P.D., Medill, S.A., Stewart, K., Andres, D., Shury, T., Wagner, B., Jenkins, E., Gilleard, J.S., Poissant, J. (2016). Negative covariance between parasite load and body condition in a population of feral horses. Parasitology 143:983-997.

Jenkins, E., Backwell, A.-L., Bellaw, J., Colpitts, J., Liboiron, A., McRuer, D., Medill, S., Parker, S., Shury, T., Smith, M., Tschritter, C., Wagner, B., Poissant, J., McLoughlin, P. (2020). Not playing by the rules: Unusual patterns in the epidemiology of parasites in a natural population of feral

horses (*Equus caballus*) on Sable Island, Canada. International Journal for Parasitology: Parasites and Wildlife 11:183-190.

Kuzmina, T., Zvegintsova, N., Zharkikh, T. (2009). Strongylid Community Structure of the Przewalski's Horses (*Equus ferus przewalskii*) from the Biosphere Reserve "Askania-Nova", Ukraine. Vestnik Zoologii 43: e-5-e-11. DOI 10.2478/v10058-009-0010-1

Kuzmina, T.A., Zvegintsova, N.S., Zharkikh, T.L. (2017). Gastrointestinal Parasite Community in a New Population of the Przewalski's Horse (*Equus ferus przewalskii*) in the Orenburg State Reserve, Russia. Vestnik Zoologii 51:243-250.

Painer, J., Kaczensky, P., Ganbaatar, O., Huber, K., Walzer, C. (2011). Comparative parasitological examination on sympatric equids in the Great Gobi "B" Strictly Protected Area, Mongolia. European Journal of Wildlife Research 57:225-232.

Pomroy, W.E., Stafford, K.J., Freeman, D.A., Grimmett, J.B., Adlington, B.C., Calder, S.M. (1995). Kaimanawa horses: preliminary parasitological results. Proceedings of the New Zealand society for parasitology, New Zealand Journal of Zoology 22:198.

Rubenstein, D.I. (2010). "Ecology, social behavior, and conservation in zebras," in Advances in the Study Behavior: Behavioral Ecology of Tropical Animals, ed. R. Macedo (Elsevier Press, Oxford, UK), 42:231-258.

Rubenstein, D.I., Hohmann, M.E. (1989). Parasites and Social Behavior of Island Feral Horses. Oikos 55: 312-320.

Seeber, P.A., Kuzmina, T.A., Greenwood, A.D., East, M.L., 2020. Effects of life history stage and climatic conditions on fecal egg counts in plains zebras (*Equus quagga*) in the Serengeti National Park. Parasitology Research 119:3401-3413.

2 Supplementary Figures

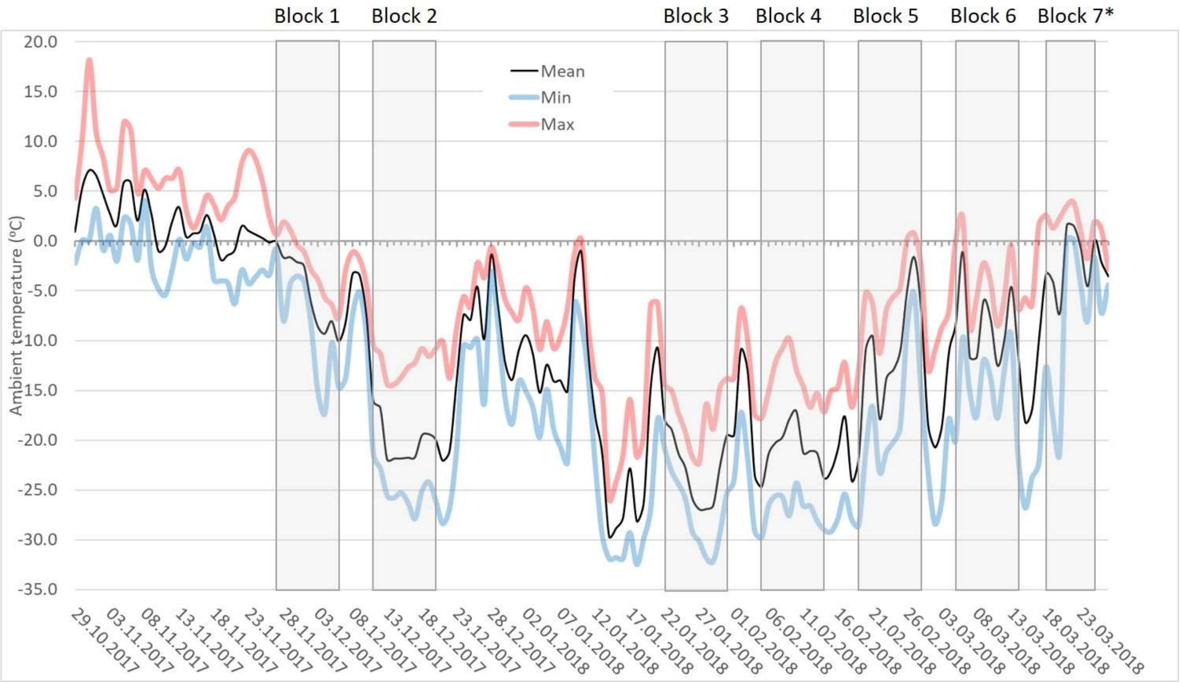

Supplementary Figure S1. Fecal sampling blocks and ambient temperature at Alibi research station on the Torgai Steppe in central Kazakhstan during winter 2017/2018. *Block 7 was 3 days shorter, stretching over 7 rather than 10 days.
